# Coincidence measurements of two quantum-correlated photon pairs widely separated in the frequency domain

**DOI:** 10.1038/s41598-023-35831-z

**Published:** 2023-05-25

**Authors:** Masayuki Hojo, Shuntaro Tani, Yohei Kobayashi, Koichiro Tanaka

**Affiliations:** 1 Department of Physics, Science, Kyoto University, Kitashirakawa-Oiwake, Sakyo, Kyoto Japan; 2 The Institute for Solid State Physics, The University of Tokyo, 5-1-5 Kashiwa-No-Ha, Kashiwa, Chiba Japan

**Keywords:** Nonlinear optics, Quantum optics, Mid-infrared photonics

## Abstract

Quantum correlation is a key concept characterizing the properties of quantum light sources and is important for developing quantum applications with superior performance. In particular, it enables photon pairs that are widely separated in the frequency domain, one in the visible region, the other in the infrared region, to be used for quantum infrared sensing without direct detection of infrared photons. Here, simultaneous multiwavelength and broadband phase matching in a nonlinear crystal could provide versatile photon-pairs source for broadband infrared quantum sensing. This paper describes direct generation and detection of two quantum-correlated photon pairs produced via simultaneous phase-matched processes in periodic crystals. These simultaneous photon pairs provide a correlated state with two frequency modes in a single pass. To confirm the correlation, we constructed an infrared-photon counting system with two repetition-synchronized fiber lasers. We performed coincidence measurements between two pairs, 980 nm and 3810 nm, and 1013 nm and 3390 nm, which yielded coincidence-to-accidental ratios of 6.2 and 6.5, respectively. We believe that our novel correlated light source with two separate pairs in the visible and infrared region complements a wide-range of multi-dimensional quantum infrared processing applications.

## Introduction

Quantum-correlated photons play an essential role in quantum technologies^1–4^. There have been attempts to develop such photon sources for quantum information and communication technology (QICT), quantum optical coherence tomography (QOCT), and quantum infrared sensing (QIS). These quantum applications have been demonstrated as a way to overcome the technical limitations in the classical experiments. One of the schemes to generate correlated photon pairs is based on a spontaneous parametric down-conversion (SPDC) process, where one photon (pump) splits into a photon pair (signal and idler) in a nonlinear crystal^5^. QICT and QOCT employ nearly degenerate processes, in which the daughter photon pairs have almost the same energy in the visible or near-infrared region. Meanwhile, under certain conditions, SPDC signals and idlers are produced in the visible and infrared region, respectively. Such widely spaced photon pairs would be useful for QIS^3,6–11^ in incompatible spectral domains, where spectral information of a target medium in the infrared region is measured without using an infrared detector, complicated infrared optics, and cryogenic systems^12^.

In QIS, it has been a pressing requirement to extend the wavelength coverage of SPDC photon pairs into the broadband infrared region. Current techniques to scan the infrared spectral range are basically performed by tuning the pump wavelength, controlling the crystal temperature, or arranging the crystal periodicity^13–17^. However, these schemes have challenges of long scanning time and poor tuning range. A fascinating method to realize the potential coverage of QIS is to construct the multiband SPDC process. The periodic structures of nonlinear crystals designed as a cascaded grating and periodically-phase-reversal grating^18,19^ serve as a discrete multiband generator with the capability for multiplexing many more frequency modes than would be possible by multiplexing using the polarization degree. So far, however, multiband SPDCs operate only at visible and near-infrared target wavelengths. In our previous study^15^, we proposed a simultaneous SPDC (s-SPDC) process in periodically poled lithium niobate (PPLN) and demonstrated the potential for a simple but multiband light source in the infrared region. In the photon-statistical context, it was pointed out that the s-SPDC photon pairs can be used to construct quantum correlated states in the frequency domain. Therefore, the s-SPDC has a possibility of being an alternative SPDC source for QIS.

One inevitable issue of infrared correlated photons is that the coincidence measurement system requires high detection efficiency and fast response for counting the number of photons in the infrared region. While fast single-photon detectors have been established in the visible light region, there are no detectors that satisfy both bandwidth and detection efficiency in the infrared region because infrared detectors would suffer from thermal noise, slow detection, or limited quantum efficiency. Up-conversion (UPC) is a way to overcome the limitations on photon-counting in the infrared region^20–22^. UPC allows infrared photons to be spectrally translated into the visible region so that they can be counted in the visible region.

In this study, we constructed an infrared photon-counting system with a pulse-gating window for distinguishing individual photons in the temporal domain, where the two correlated photon pairs are produced via the s-SPDC process and translated into visible light by the up-converter. The light sources for s-SPDC and UPC are based on a repetition-synchronized fiber-based mode-locked laser system. The UPC system converts the idlers into visible photons with a conversion efficiency of 29%. We implemented coincidence measurements and estimated the coincidence to accidental-coincidence ratios (CAR), which is the experimental index to evaluate the purity of photon correlation. Our results indicate that the s-SPDC method can be used to make a multi-wavelength nonlinear down-converter with strongly correlated states.

## Results

### Synchronized operation of s-SPDC and UPC

**Figure 1:**
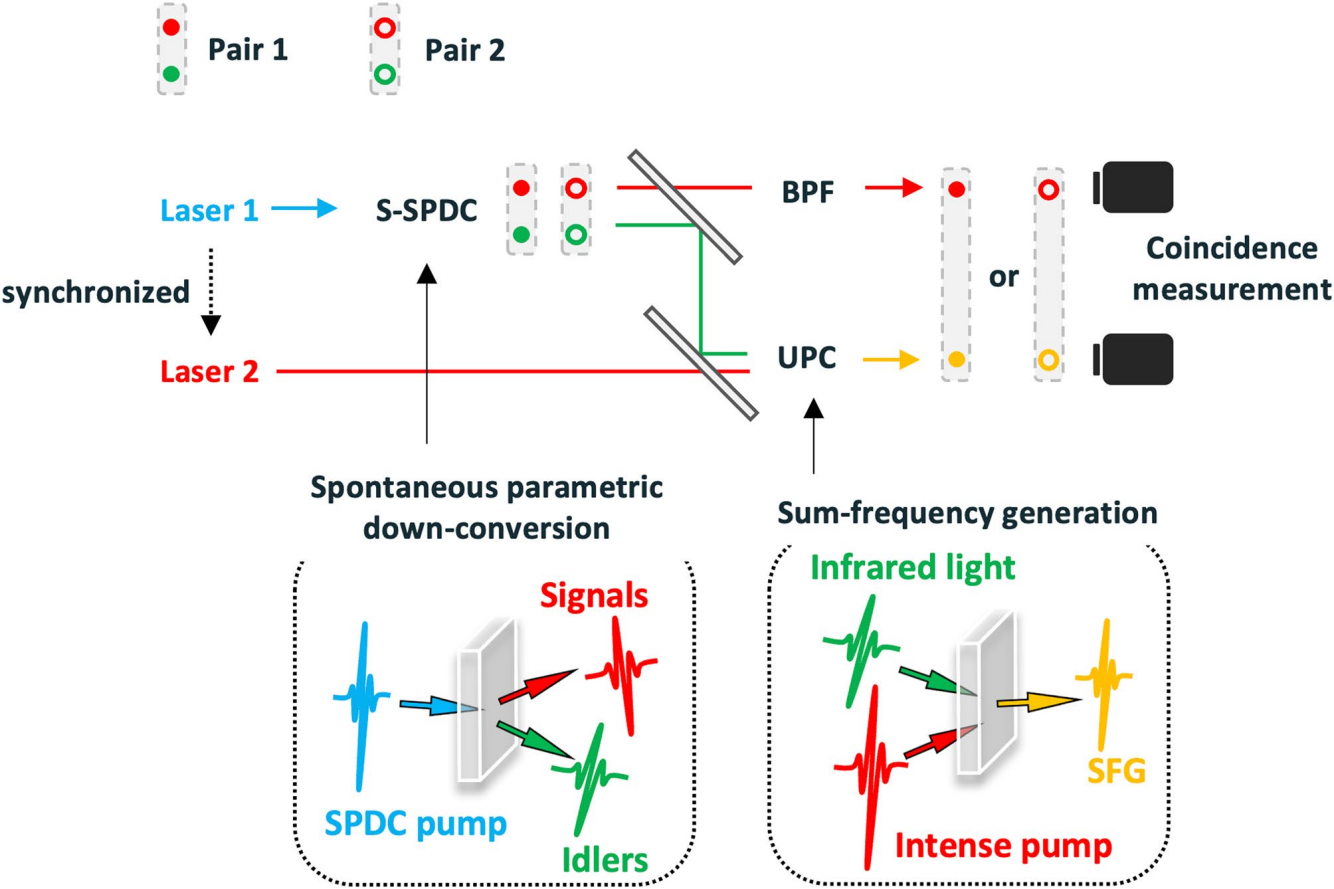
Conceptual diagram of two correlated photon pairs produced by simultaneous SPDC processes. The infrared idlers of the pairs are introduced into the up-converter (UPC) and translated into the visible region. The corresponding signals are selected by the band-pass filter (BPF). The signal and idler pairs maintain their correlation even after UPC.

Figure 1 shows a conceptual diagram of the s-SPDC generator and UPC detector. Measuring the correlation of photon pairs requires temporally well separated photons in excess of the dead time of photon counting detectors. It follows that pulsed, not continuous-wave, pumps are suitable for exciting the s-SPDC process. An UPC detector pumped by a pulse laser is a significant technique to achieve high detecting efficiency of infrared photons. Moreover, a fiber-based mode-locked laser system provides an effective way to prepare stable and high-power pulse sources for both processes. Therefore, we prepared repetition-synchronized lasers which can be used for generating and detecting the SPDC pairs.

The s-SPDC process takes place with the pump wavelength in the range of 600–900 nm in the PPLN^15^. Especially, a spectral range of 2–5 µm for the idlers, which is of a great importance for infrared spectral measurements, can be covered by a pump at around 780 nm. Therefore, we used a frequency-doubled erbium-doped fiber laser (EDFL) as a s-SPDC pump. In contrast, the UPC pump pulse was generated by an ytterbium-doped fiber laser (YDFL) oscillating at 1030 nm in order to amplify efficiently the pulse intensity with high-gain ytterbium-doped double-clad fibers.

The output wavelengths of the s-SPDC pairs were simulated under the quasi-phase-matching (QPM) condition:

1 $$\Delta K=\left|\overrightarrow{{K}_{1}}-\overrightarrow{{K}_{2}}-\overrightarrow{{K}_{3}}-\overrightarrow{{G}_{\Lambda }}\right|=0$$

**Figure 2:**
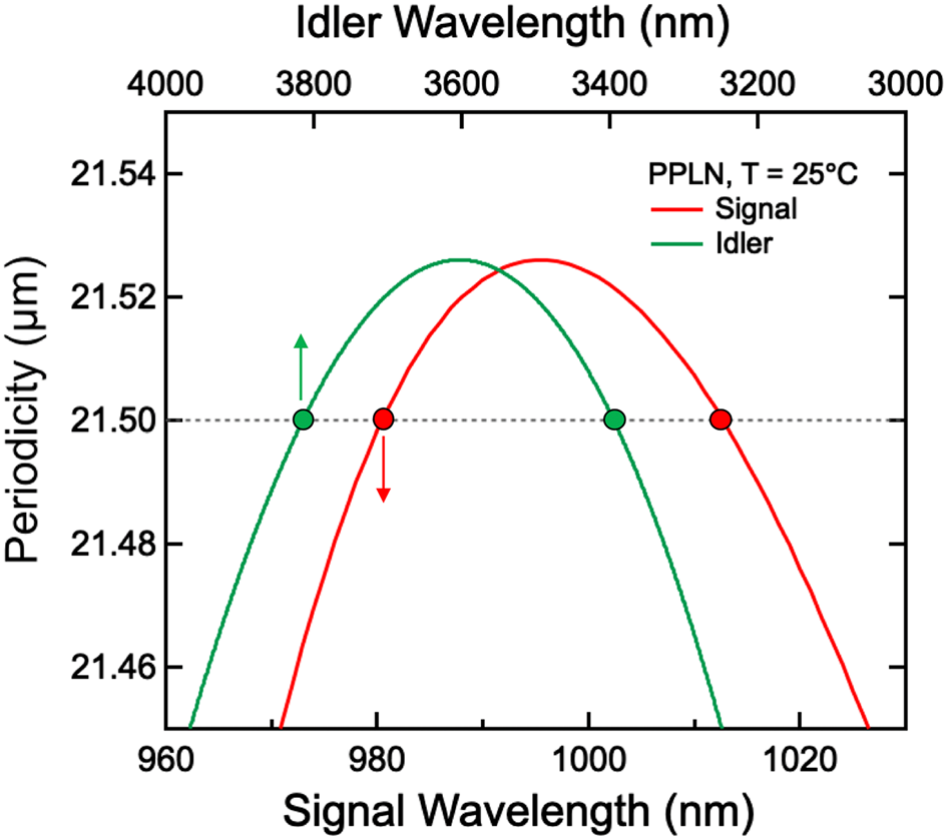
Theoretical calculation of collinear quasi-phase-matching condition for 780-nm pump laser as a function of the periodicity of the PPLN crystal (vertical axis). The bottom axis represents the SPDC signals (red curve) and the top the SPDC idlers (green curve). In the case of a periodicity of 21.5 µm, two pairs (Pair 1: 980 nm and 3810 nm; Pair 2: 1013 nm and 3390 nm) are simultaneously quasi-phase-matched under the same conditions.

Here, $\left|\overrightarrow{{K}_{i}}\right|=2\pi n\left({\lambda }_{i},T\right)/{\lambda }_{i}$ denotes the wave vectors of the pump ($i$=1), idlers ($i$=2), and signals ($i$=3). $\left|\overrightarrow{{G}_{\Lambda }}\right|=2\pi /\Lambda$ is the superlattice vector obtained from the periodicity Λ for compensating the phase mismatch. $n\left({\lambda }_{i},T\right)$ is the refractive index of the crystal at wavelength ${\lambda }_{i}$ and temperature $T$. Figure 2 shows the phase-matched wavelengths of signals and idlers for various periodicities in the s-SPDC process in PPLN pumped at 780 nm and at T = 25℃. The plotted values were calculated using the temperature-dependent Sellmeier equation^23^. In the case of a periodicity of 21.5 µm (the dashed line), two pairs, 980 nm and 3810 nm (pair 1) and 1013 nm and 3390 nm (pair 2), were simultaneously generated in a collinear direction. In order to detect the signals in Si photon counting detector (400–1100 nm), we chose a periodicity of 21.5 µm. Note that the non-collinearly phase-matched signals (idlers) covered the spectral range between the two collinear signals (idlers)^15^. For the periodicity of 21 µm, the non-collinear idlers within the radiated angle of 5 degrees covered the infrared range of 2–5 µm. These results show that the non-collinear SPDC pairs can function as a broadband quantum light source with multi-dimensional correlation.

### Specifications of the UPC and SPDC pumps

**Figure 3:**
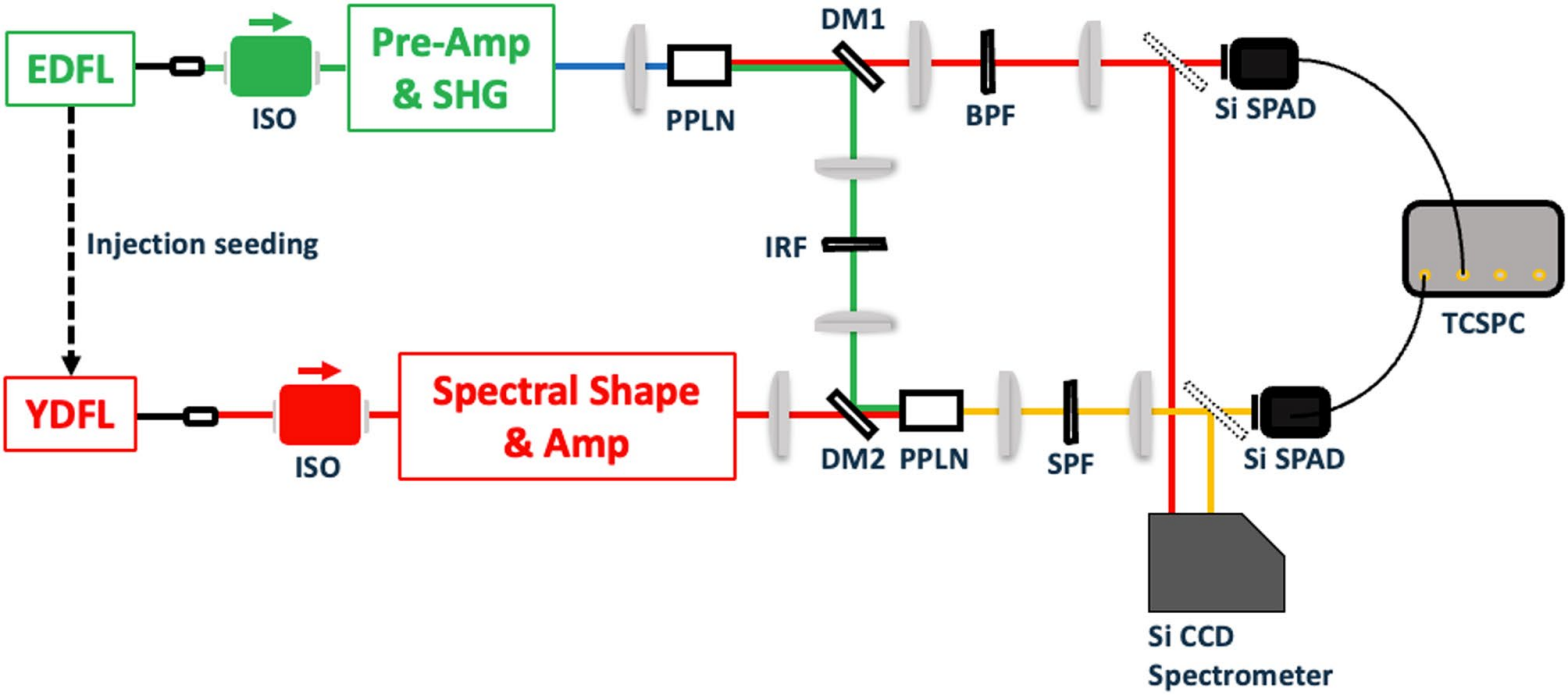
Schematic layout of the experiment. *EDFL* erbium doped mode-locked fiber laser, *YDFL* ytterbium doped mode-locked fiber laser, *ISO* isolator for removing back-reflecting light, *Pre-Amp* amplifying system using erbium doped fiber, *SHG* second harmonic generation port using a PPLN crystal, *DM1* dichroic mirror for separating signals (transmitted) and idlers (reflected), *BPF* band pass filter for cutting SPDC pump and extracting the corresponding signals, *IRF* infrared pass filter. Spectral Shape: Chirped fiber Bragg grating with 0.6-nm reflection band. Amp: Amplifying system using ytterbium-doped double-clad fiber and grating pairs for compensating its dispersion. *DM2* Dichroic mirror for coupling the idlers and the UPC pump. *SPF* short pass filter for cutting the UPC pump, *Si SPAD* Si-based single photon counting avalanche photodiode, *TCSPC* Time correlated single photon counting module.

**Figure 4:**
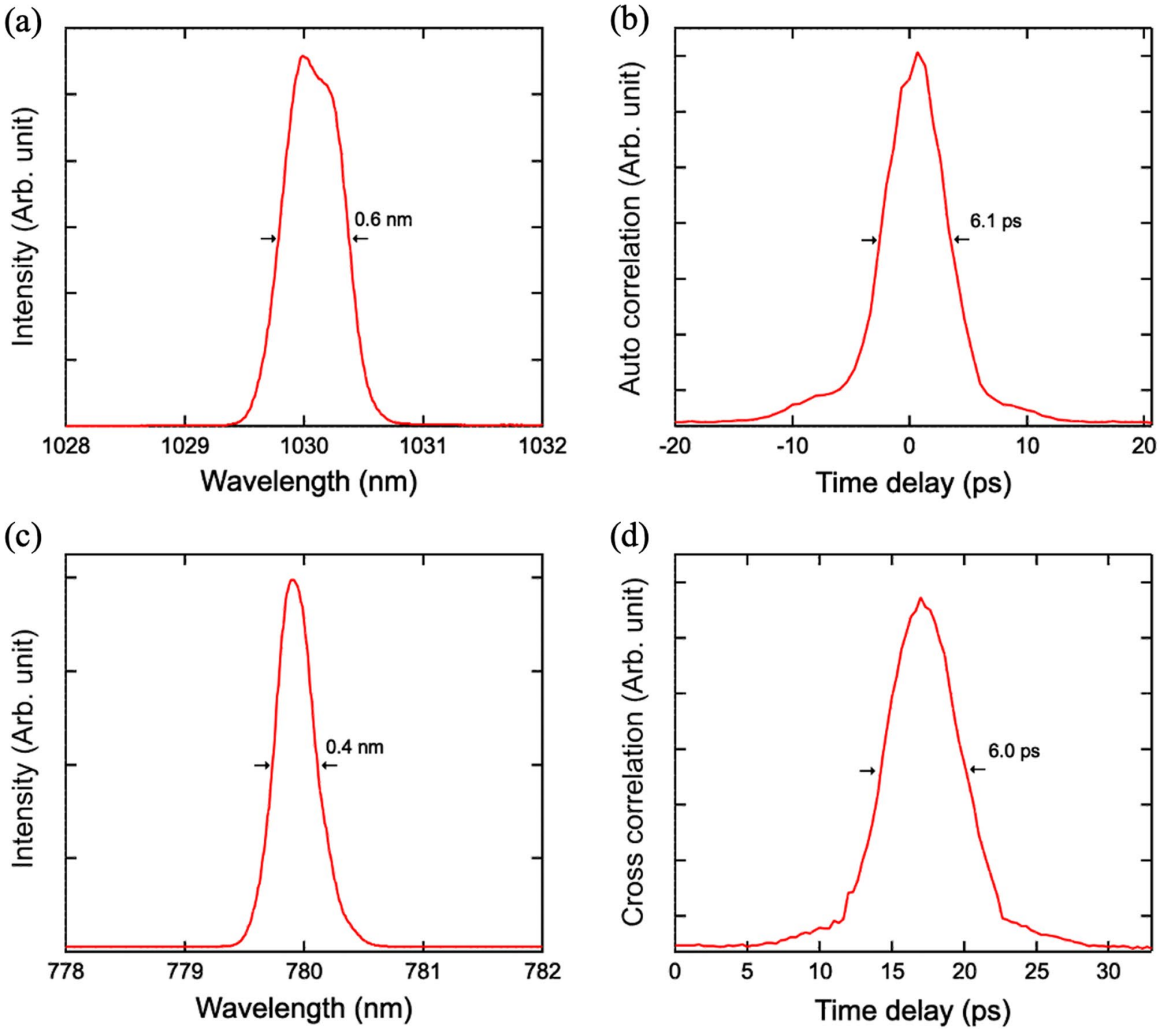
Specification of the UPC and s-SPDC pump. (**a**) Spectrum of the UPC pump pulse. The spectral FWHM width is 0.6 nm. (**b**) Auto-correlation curve of the UPC pump pulse. A scaling factor of $\sqrt{2}$ was used to deduce the temporal duration to be 4.3 ps under the assumption of a Gaussian profile. (**c**) Spectrum of SPDC pump pulse produced by SHG process of the Er pulse. The spectral FWHM width is 0.6 nm as well. (**d**) Cross-correlation curve between the UPC and SPDC idler pulses. The active duration of the idlers was estimated to be 4.1 ps under the assumption of a convolution between Gaussian profiles.

We constructed a synchronized laser system composed of two fiber-based oscillators. The experimental setup is illustrated in Fig. 3. The two fiber-based lasers were repetition-synchronized by injecting the EDFL pulse into the YDFL cavity^24^. The EDFL system for pumping the s-SPDC is combined with the YDFL system for UPC by injecting the EDFL pulse into the YDFL oscillator. Cross-phase modulation between the pulses enabled synchronous repetition at 79.6 MHz. (The details of the laser sources are explained in Supplementary 1) The EDFL pulse is divided into two portions, which respectively serve as the master laser for synchronization and as the s-SPDC pump after SHG. Figure 4c shows the specifications of the SPDC pump. The s-SPDC pump is generated at 780 nm with 10 mW average power and a 0.4 nm bandwidth. Note that the s-SPDC pump was attenuated to less than 1 mW in coincidence measurements in order to suppress the accidental events. The details of optimizing the pump power are explained in Supplementary 2. The spectral and temporal full width at half maxima (FWHM) of the UPC pump are 0.6 nm and 4.3 ps as shown in Fig. 4a,b, which are 1.7 times those of a Fourier-transformed pulse. Moreover, as shown in Fig. 4d, we measured cross-correlation between the UPC pump and idlers in order to estimate the temporal profile of the idlers, with an FWHM of 6.0 ps. It means that a pulse duration of the incident idlers should be 4.1 ps, which is comparable to the temporal width of the UPC pump. According to the theoretical analysis, the conversion efficiency reduces by 30% because of such a temporal mismatch between the UPC pump and idlers. The estimation details are shown in Supplementary 3.

### Specifications of the up-converter

In order to verify that the UPC detector is useful for detecting the idlers, we theoretically estimated the conversion efficiency and experimentally demonstrated its validity. According to the theoretical simulation of s-SPDC, the total flux of the idlers is around ${10}^{7}$ photons/s in the collinear SPDC process with the pump average power 10 mW. It is difficult to detect such a microscopic output flux by a broad-bandwidth photodetector. Moreover, the conversion efficiency must be sufficiently high to preserve the quantum information after UPC. Using an intense pulse with a narrowband and short-temporal duration as the UPC pump attains high conversion efficiency^22,25^ and allows the number of the weak SPDC idler photons to be counted with sensitive detectors such as a Si-based single photon avalanche photodiode (Si-SPAD). The conversion efficiency $\Phi$ from the infrared idler with a center frequency at ${\omega }_{2}$ to the visible photon at ${\omega }_{3}$ is expressed as follows^25^:

2 $$\Phi =2{Z}_{0}{\gamma }^{2}{I}_{1}{L}^{2}{\mathrm{sinc}}^{2}\left[L{\left\{{\left(\frac{\Delta k}{2}\right)}^{2}+2{Z}_{0}{\gamma }^{2}{I}_{1}\right\}}^\frac{1}{2}\right],$$

where

$$\gamma =\frac{{d}_{eff}}{c}\sqrt{\frac{{\omega }_{2}{\omega }_{3}}{{n}_{2}{n}_{3}}} \mathrm{and} \Delta k=\left|\overrightarrow{{k}_{1}}+\overrightarrow{{k}_{2}}-\overrightarrow{{k}_{3}}+\overrightarrow{{G}_{\Lambda }}\right|,$$

and where $\left|\overrightarrow{{k}_{i}}\right|=2\pi n\left({\lambda }_{i},T\right)/{\lambda }_{i}$ denotes the wave vector of the pump ($i$=1), infrared ($i$=2) and up-converted ($i$=3) light. ${Z}_{0}$ is the impedance of free space, ${I}_{1}$ [W/m^2^] is the peak intensity of the UPC pump, and $L$ is the interaction length in the nonlinear crystal. ${d}_{eff}$ [pm/V] is the effective nonlinear coefficient reduced by the periodic structure. The simulated efficiency at the maximum UPC pump power 190 mW was estimated to be 61%. In addition, as Eq. (2) indicates, the phase-matched wavelengths cover a certain range depending on the pump spectral width and the crystal length. For a spectral width of 0.6 nm, the UPC windows were calculated to be 29.3 nm for translating the infrared light at around 3810 nm, which corresponded to 1.95 nm for the accompanying signals. We describe the spectral and temporal windows of the UPC detector in Supplementary 4. In our experimental setup, the UPC efficiency at the maximum UPC pump power 190 mW was estimated to be 29% in a 29.3 nm spectral window. Here, the maximum pump power is restricted by self-phase-modulation (SPM) in the amplifying fiber because SPM broadens the spectral and temporal profiles of the UPC pulse. Therefore, SPM reduces the peak intensity and the conversion efficiency in turn. The details of the efficiency estimation are explained in Supplementary 3.

### Spectral filtering for extracting accompanying pairs

To measure the correlation of the s-SPDC photons, the temporal timing and spectral width of the signals must be identical to those of the up-converted idlers. For the temporal timing, the group velocity mismatch between the signals and idlers in the PPLN should be less than the repetition period 12.5 ns of the UPC pump pulse train. The temporal delay between 980 and 3810 nm induced in a 20-mm-long crystal is calculated to be 3.1 ps, which is much shorter than the repetition period 12.5 ns. However, the spectral range of the signals needs post-filtering due to the non-collinear phase-matched SPDC photons and the tolerant bandwidth of the up-converter. As described above, the UPC process also functions as a bandpass filter with a 29.3-nm-wide converting band. Therefore, extracting only the corresponding signals is required.

**Figure 5:**
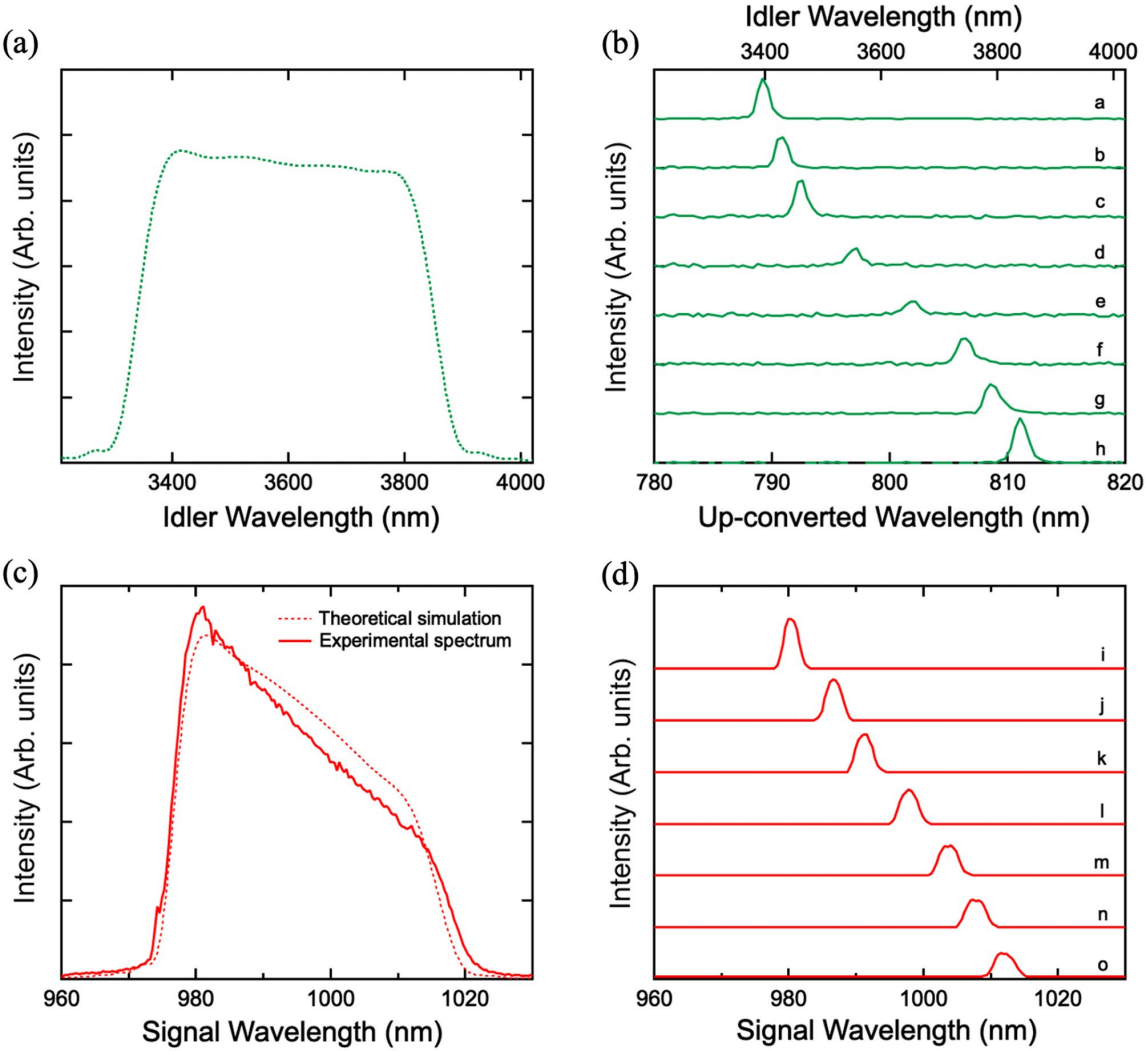
Simulated and experimental spectra of the s-SPDC signals and idlers. (**a**) Simulated spectrum of the idlers including the non-collinear components. The radiated angle of 0.8 degree covered the range of 3390–3810 nm. (**b**) Spectra of up-converted idlers at different crystal temperatures and periodicities in which the idlers were phase-matched at different output angles. a: 21.5 µm and 60℃. b: 21.5 µm and 80℃. c: 21.5 µm and 100℃. d: 21.8 µm and 60℃. e: 21.8 µm and 100℃. f: 22.1 µm and 60℃. g: 22.1 µm and 80℃. h: 22.1 µm and 100℃. Simulation conditions a and h correspond to the collinear configuration. (**c**) Theoretical simulation and experimental spectrum of the total signals including the non-collinear configuration with covered range of 980–1013 nm. (**d**) Spectra of extracted signals using a narrow band-pass filter. The tolerant bandwidths were selected by tilting the filter. Simulation conditions i and o correspond to the collinear configuration.

First, we compared simulated and experimental spectra of the up-converted idlers. Figure 5a shows the spectrum of idlers simulated using the equation of the average photon number ${F}_{2}$ [Hz] of the idlers as follows^15^:

3 $${F}_{2}=\int d{\omega }_{2}d{\theta }_{2}\frac{{\omega }_{2}^{3}{\omega }_{3}{n}_{2}^{2}{L}^{2}{d}_{eff}^{2}{P}_{1}}{2{\pi }^{2}{c}^{5}{\varepsilon }_{0}{n}_{1}{n}_{3}}\frac{\mathrm{sin}{\theta }_{2}}{{\mathrm{cos}}^{3}{\theta }_{2}}\mathrm{ Sin}{\mathrm{c}}^{2}\left[\frac{\Delta KL}{2}\right].$$

Here ${\theta }_{2}$ is the output angle of the idlers against the collinear direction, and ${P}_{1}$ [W] is the SPDC pump power. The non-collinear components within the radiated angle of 0.8 degree covered a range of 3390–3810 nm. Considering such broadband coverage, we extracted the spectra at the different crystal temperatures and periodicities in which the idlers were phase-matched at different output angles, as shown in Fig. 5b. The two outermost peaks (conditions a and h) corresponded to the collinear pairs indicated by the green markers in Fig. 2. The intensity of the non-collinear idlers (conditions b-g) was smaller than that of the collinear ones because of the mismatch of the spatial mode and shrinking of the interaction length in the nonlinear crystal. The spectral widths of the up-converted collinear idlers in the infrared region were 29.3 nm for 3810 nm and 19.8 nm for 3390 nm; these values are in good agreement with the theoretical UPC window. The accompanying signals corresponded to spectral widths of 1.95 nm for 980 nm and 1.85 nm for 1013 nm.

Second, we controlled the signal spectra. The dashed curve in Fig. 5c is the theoretical simulation of a broad non-collinear spectrum with a coverage range of 980–1013 nm. It fairly closely matches the experimental curve indicated by the solid curve. In order to suppress noise counts in the coincidence measurements, we extracted only the signal components corresponding to the up-converted idlers by using a narrow band-pass filter. The spectra of the extracted signals are shown in Fig. 5d. Again, the two outermost peaks (conditions i and o) corresponded to the collinear pairs indicated by the red markers in Fig. 2. The non-collinear components were measured by changing the angle of the filter. The widths of the signals were estimated to be 2.4 nm for 980 nm and 3.3 nm for 1013 nm. This mismatch reduced the purity of the correlation by a few percent.

### Coincidence measurement of simultaneous SPDCs

**Figure 6:**
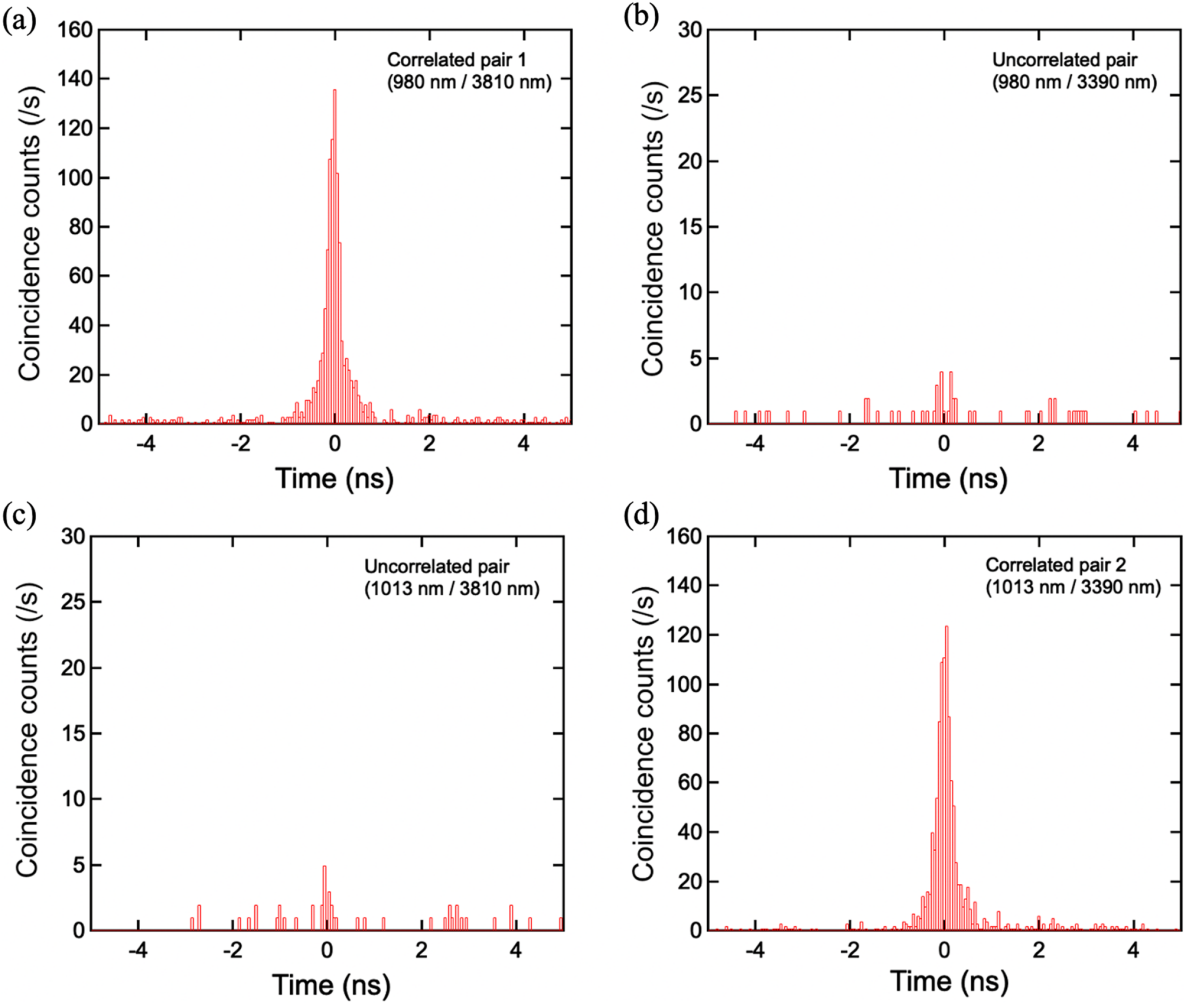
Coincidence histograms of (**a**) correlated pair 1, 980 and 3810 nm, (**b**) uncorrelated pair, 980 and 3390 nm, (**c**) uncorrelated pair, 1013 and 3810 nm, and (**d**) correlated pair 2, 1013 and 3390 nm. (a) and (d) show the clear peak within a temporal window of 1 ns. Coincidental noise counts were accidentally obtained in (b) and (c).

We performed coincidence measurements on the filtered signals and up-converted idlers. The coinciding events were logged in the TCSPC module as a function of the time delay between the two detectors when both Si-SPADs detected photons within a 50-ps temporal bin, much shorter than the system temporal resolution 1 ns. In order to compare the numbers of coincident events for the correlated and uncorrelated pairs, the temporal window was determined by the active width of the coincidence histogram. We performed the measurements for relevant four pairs taken from either of two signals (980 nm and 1013 nm) and either of two idlers (3810 nm and 3390 nm). Figures 6a and 6d show typical histograms of coincident count s of the two correlated pairs with the SPDC pump power 0.93 mW, in which clear peaks were observed in a temporal window of 1 ns. In contrast, the histograms of the two uncorrelated pairs exhibited little counts in Fig. 6b,c. These four histograms show that each pair 1 coincidence originates from a single pump photon, and, separately, each pair 2 coincidence originates from a (different) single pump photon. To theoretically investigate this quantum state, we depicted the total quantum state produced via a single pulse $|\psi \rangle$ as follows:

4 $$|\psi \rangle =\left\{\sum_{k\ge 0}{c}_{k}{\left({\mu }_{1}\right)}^{k}\left|{k\rangle }_{{\omega }_{s1}}\right|{k\rangle }_{{\omega }_{i1}}\right\}\left\{\sum_{i\ge 0}{{c}_{i}\left({\mu }_{2}\right)}^{i}\left|{i\rangle }_{{\omega }_{s2}}\right|{i\rangle }_{{\omega }_{i2}}\right\}$$

Here $|{n\rangle }_{{\omega }_{s(i)j}}$ is the n-number SPDC state of the signal (idler) of the pair j (j = 1,2) with center frequency ${\omega }_{s(i)j}$. ${\left|{\mu }_{1\left(2\right)}\right|}^{2}$ is the SPDC efficiency of pair 1 (2) and ${C}_{n}={c}_{m}{c}_{n-m}$ ($0\le m\le n$) is a constant factor for normalizing each state. Expanding the Eq. (4) for both k and i, we obtained the quantum states describing all possible processes. The first is ${C}_{0}\left|{0\rangle }_{{\omega }_{s1}}\right|{0\rangle }_{{\omega }_{i1}}\left|{0\rangle }_{{\omega }_{s2}}\right|{0\rangle }_{{\omega }_{i2}}$ ($k=i=0$), which corresponds to the residual pump light itself without any SPDC pairs. The second is ${C}_{1}\left({\mu }_{1}\left|{1\rangle }_{{\omega }_{s1}}\right|{1\rangle }_{{\omega }_{i1}}\left|{0\rangle }_{{\omega }_{s2}}\right|{0\rangle }_{{\omega }_{i2}}+{\mu }_{2}\left|{0\rangle }_{{\omega }_{s1}}\right|{0\rangle }_{{\omega }_{i1}}\left|{1\rangle }_{{\omega }_{s2}}\right|{1\rangle }_{{\omega }_{i2}}\right)$, which is the simultaneous SPDC state that we focus on in our experiment. The coincidence counts shown in Fig. 6a,d are attributed to this state. The third is the case of the higher-number SPDC events included in one pulse such as ${C}_{2}\left({\left({\mu }_{1}\right)}^{2}\left|{2\rangle }_{{\omega }_{s1}}\right|{2\rangle }_{{\omega }_{i1}}\left|{0\rangle }_{{\omega }_{s2}}\right|{0\rangle }_{{\omega }_{i2}}+{\mu }_{1}{\mu }_{2}\left|{1\rangle }_{{\omega }_{s1}}\right|{1\rangle }_{{\omega }_{i1}}\left|{1\rangle }_{{\omega }_{s2}}\right|{1\rangle }_{{\omega }_{i2}}+{\left({\mu }_{2}\right)}^{2}\left|{0\rangle }_{{\omega }_{s1}}\right|{0\rangle }_{{\omega }_{i1}}\left|{2\rangle }_{{\omega }_{s2}}\right|{2\rangle }_{{\omega }_{i2}}\right)$, which means that two SPDC pairs simultaneously generated from a single pulse. Note that ${\mu }_{1\left(2\right)}$ is basically less than ${10}^{-9}$ in the unsaturated pump regime, which follows that the probability with two or more pairs generated from a single pulse should be significantly suppressed. Therefore, the higher-number SPDC pairs rarely contributed to accidental coincidence logged in the uncorrelated pairs as shown in Fig. 6b,c. Rather, accidental coincidence is attributed to the excess SPDC pairs and the up-converted noise photons from the UPC pump itself^26^. The characterization of the accidental events is given in Supplementary 2.

Moreover, we measured accidental-coincidence counts that were outside the window to calculate the coincidence to accidental-coincidence ratios, CAR:

5 $$CAR=\frac{{C}_{tr}}{{C}_{ac}}=\frac{{C}_{to}-{C}_{ac}}{{C}_{ac}}.$$

**Figure 7:**
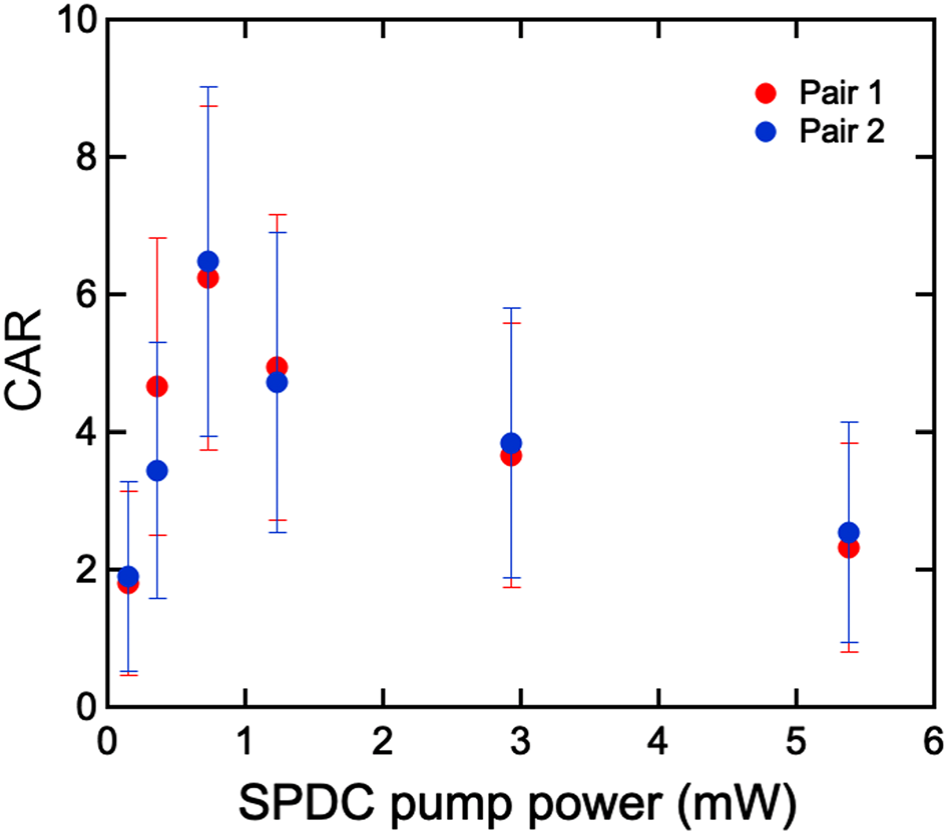
Coincidence-to-accidental coincidence ratios against SPDC pump power over an integration period of 1 s. In the range of low pump power, the number of SPDC pairs is not sufficient for overcoming the total noise counts in the whole system. In contrast, high pump power supplies a rate of pair generation in excess of the dead-time rate (~ 78 ps) of the SPADs. At a maximum CAR of 6.2 and 6.5, the number of true coincidence events were 123 and 136, whereas the number of accidental events were 17 and 21, within an integrated period of a second. The error bars are derived from the standard deviation of the true coincidence and accidental backgrounds.

We investigated accidental events ${C}_{ac}$ between the signals (idlers) and uncorrelated photons by delaying the counting window by a cycle of the laser repetitions lasting 12.5 ns. The number of true coincidence counts ${C}_{tr}$ was calculated by subtracting ${C}_{ac}$ from the total counts ${C}_{to}$. Figure 7 plots CAR as a function of the SPDC pump power. At an average power of 0.93 mW, the maximum CAR values of 6.2 and 6.5 were attained for SPDC pair 1 and 2. These values were limited by the number of the uncorrelated photons produced by the mismatch of filtering or the total quantum efficiency including the detector sensitivity, and were relatively smaller than values determined in coincidence experiments using a pulsed UPC or cavity-type UPC^20,21^. The purity of the correlation is a fundamental parameter specifying the availability of correlated pairs for QICT or QIS. Here, there is room for improving the CAR by optimizing the pump wavelength for generating the signals in more sensitive detecting range of Si-SPAD. For instance, sum-frequency generation (SFG) of the YDFL (1030 nm) and EDFL (1560 nm) allows to prepare the pump (620 nm). Moreover, we can radically improve the measured CAR value by employing detectors that are more sensitive in the near-infrared region covering the signal wavelengths around 1000 nm. In the present setup, the quantum efficiency of the Si-SPAD is around 3% for signals at around 1000 nm. The poor quantum efficiency of SPADs significantly decreases the coincidence counts, so that the SPDC pump power should be enhanced up to clearly distinguish the true coincidence from dark counts or accidental noises. However, high power SPDC pump power produces finite accidental noises when the SPDC pair rate is comparable to the laser repetition 79.6 MHz. Therefore, using the efficient detector allows to use lower SPDC pump power, which suppresses the accidental counts to improve the CAR values. Additionally, the use of a proper band-pass filter for strictly extracting the corresponding SPDC pairs or a single-mode fiber coupling system for introducing the photons into SPAD can reduce uncorrelated photons radiating in non-collinear directions.

## Conclusion

Correlation measurements on s-SPDC pairs were performed using a UPC photon counter. The counting system was based on a nonlinear up-converter PPLN and repetition-synchronized mode-locked fiber lasers. We obtained a strong correlation only between the corresponding pairs of the visible signal and the infrared idler. This is an essential result not only for demonstrating multi-wavelength SPDC pairs in a quantum-correlated state but also for indicating the application of generating entangled photon pairs based on the multi-frequency modes using multiple phase-matching conditions^18^, which can be employed in spectroscopy using coincident events between photon pairs^27,28^.

Our technique can be further developed into one for generating multi-dimensional photon pairs using the non-collinear configuration. Under optimal conditions for s-SPDC in terms of coverage of the infrared range, the idlers can cover 2–5 µm within 5 degrees of the output angle, whereas the corresponding signals can cover 0.92–1.27 µm within 1 degree. The most useful aspect of this light source is that although the infrared photons themselves are uncorrelated, every pair of visible and infrared photons is strongly correlated even if the light source involves many components such as unexpected noise, higher-number down-conversion events or imperfect mode overlap which contribute to the measured CAR values which are not perfectly correlated. This significant capability will propel the development of multi-dimensional quantum sensing and spectroscopy by employing the more broadband UPC window, where the UPC pump is tightly focused into a PPLN in order to extend the phase-matching range in the non-collinear configuration^29^. For near-future applications, integrating the multi-wavelength technique into quantum communications or processing would be a robust way to extend the availability of quantum platforms into the well-discrete infrared range.

## Methods

### The experimental setup

The s-SPDC pump was focused into a 20-mm-long PPLN crystal. The output signals are collimated and focused into an Si-SPAD or Si-CCD spectrometer after the signals and idlers are separated by dichroic mirror 1 (DM1). The idlers reflected by DM1 pass through an infrared-pass filter in order to remove the residual pump after they are collimated by an off-axis parabolic mirror lens and are then introduced into the UPC port. Before the UPC port, the YDFL pulse is amplified in a chirped-pulse-amplification system^30^ for restricting the broadening effect of self-phase-modulation in the amplifying fiber. The system is composed of a chirped fiber-Bragg grating with a 0.6-nm-wide reflective band, an amplifier formed from ytterbium-doped double-clad fibers excited by a high-power diode laser, and grating pairs for compressing the temporal chirp. The output average power is boosted to about 190 mW. The idlers and UPC pump are spatially and temporally coupled by dichroic mirror 2 (DM2) and focused into another PPLN crystal. After the PPLN, the pump is removed by short-pass filters and only the up-converted idler is introduced into the Si-SPAD or Si-CCD spectrometer. The time resolution of the entire coincidence measurement system, including the time correlated single photon counter (TCSPC) module, was 1 ns.

## Supplementary Information

Supplementary Information.

## Data Availability

All the raw and processed data used in the figures in the main text and Supplementary Information are available in Zenodo repository (https://zenodo.org/record/7609179#.Y-C-WOzP23I).
