# Supplementary material for: Coincidence measurements of two quantum-correlated photon pairs widely separated in the frequency domain
Source: Sci Rep. 2023 May 25;13:8520. doi: 10.1038/s41598-023-35831-z (PMC10213056; doi:10.1038/s41598-023-35831-z)
Supplement: Supplementary file 1 — Supplementary Information. [file 41598_2023_35831_MOESM1_ESM.pdf]

Supplementary Information

Coincidence measurements of two quantum-correlated photon-pairs widely separated in the frequency domain

Masayuki Hojo*, Shuntaro Tani, Yohei Kobayashi and Koichiro Tanaka*

1. Synchronized fiber laser system

We explain the whole setup and specification of the fiber-based laser system. Figure S1(a) shows the experimental setup of the synchronized fiber laser system. Our system was composed of erbium- and ytterbium-doped fiber lasers (EDFL and YDFL). Both cavities were designed in ring types, including a laser diode for exciting the erbium- or ytterbium-doped fiber (ErDF and YbDF), a wavelength division multiplexing (WDM) for mixing the pump light of the laser diode with a spontaneous emission from the ErDF (or YbDF) and an output port of a polarization beam splitter[1,2]. Mode-locked pulses are generated from the output port under a given condition of the fiber length and the retardation of the wave plates. We chose the EDFL as a master cavity for repetition-synchronization. The repetition rate of the EDFL was measured as 79.581 MHz. A portion of the Er pulse was introduced into the injection port of the YDFL cavity (the slave cavity) after being amplified to the average power of 80 mW. The YDFL cavity includes the temporal delay stage for tuning the mismatch of the cavity length. When the repetition of the YDFL is near to that of the EDFL, two cavities oscillate with the same repetition using cross-phase modulation[3,4]. As shown in Fig. S1(b), there is a certain range of cavity-tuning with a delay tolerance range of 24 μm. In order to maintain the synchronized condition, the central wavelength of the YDFL was shifted by tuning the slave cavity lengths.

(a)

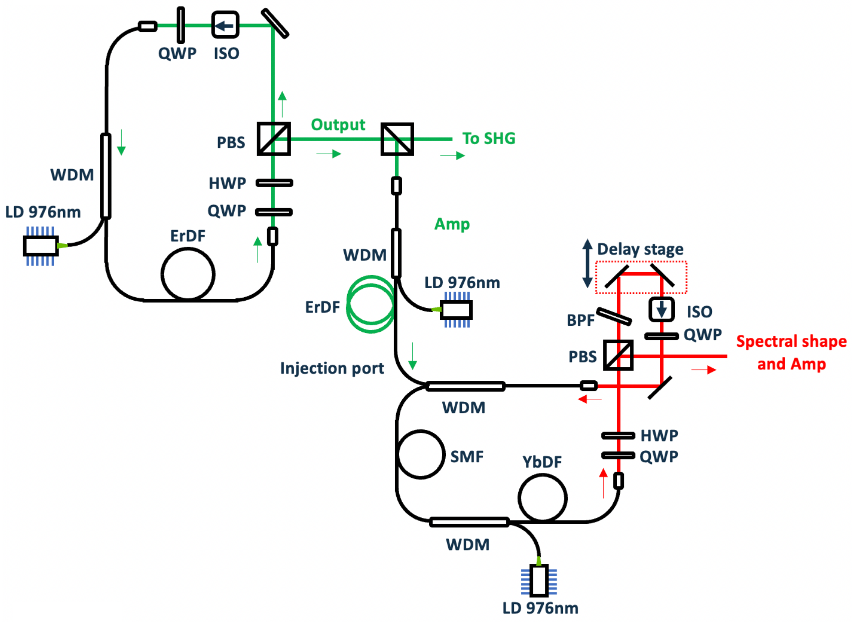

(b)

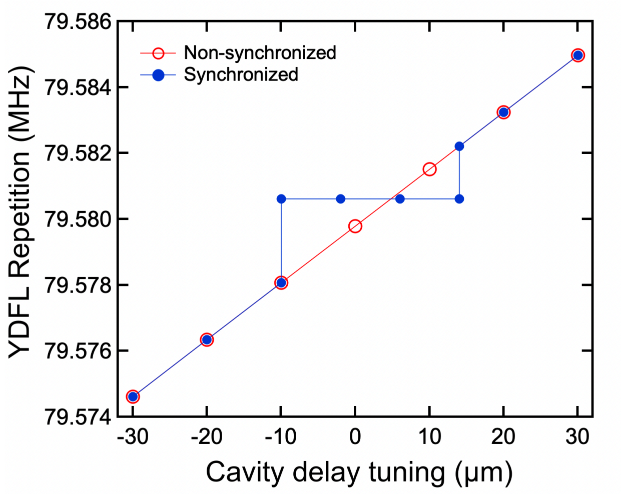

Fig. S1(a) Schematic layout of the two synchronized oscillators. ErDF: Erbium-doped fiber. YbDF: Ytterbium-doped fiber. SMF: Single mode fiber. WDM: Wavelength division multiplexing for mixing the pump diode laser with cavity seeding light. HWP: Half wave plate. QWP: Quarter wave plate. ISO: Isolator. BPF: Band pass filter for compensating the dispersion induced in the fiber. PBS: Polarization beam splitter. Amp: Amplifying system using erbium doped fiber excited by a laser diode. Injection port: The Er pulse is introduced into the Yb cavity using the WDM. (b) Repetition rates of the YDFL under different cavity length mismatches. The red curve is without injecting the EDFL pulse, and the blue one is with the EDFL pulse. The synchronized repetition rate is 79.581 MHz.

2. Background counts of the uncorrelated photons

We estimate the background noises produced via uncorrelated photons. Because of the 1-ns pulse gating, the intrinsic dark counts of the Si-SPAD and background radiation are negligible. A large part of uncorrelated counts is attributed to two factors. The first is the excess power of the SPDC pump. As described in the main manuscript, enhancing the SPDC power is required for the low efficiency of SPADs, which contributed to the accidental coincidence produced between each pulse. The second is the up-converted SPDC (USPDC) noise[5]. In the UPC crystal, the UPC pump excites not only the sum-frequency-generation but also the SPDC process which is phase-matched with the UPC pump wavelength. This SPDC light can be up-converted by the identical pump pulse. Figure S2(a) shows the SPDC pair rate per pulse. As expected for the spontaneous process, the rate of a pair of SPDC is proportional to the SPDC pump power. Figure S2(b) shows the true and accidental coincidence as a function of the SPDC pump power. The true coincidence C_{tr} is proportional to the SPDC pump power. In contrast, the accidental coincidence C_{ac} can be described as a product of single count of each port as follows:

$$C_{ac} = \frac{(S_s + N_s)(S_i + N_i)}{R_{rep}} \qquad (S1)$$

Here R_{rep} is the repetition of the synchronized laser system, which is 79.6 MHz. $S_{s(i)}$ is the number of the single count in the signal (idler) detecting port and $N_{s(i)}$ is independent noise sources in the signal (idler) detecting port such as USPDC or dark counts of the APD detectors. The equation (S1) indicates that the accidental coincidence quadratically depends on the pump power. The numerical value of the accidental counts using each count shown in Fig. S3(a) well reproduces the experimental data points as shown by the green dashed line and circle in Fig. S2(b). The generation rate should be even lower than the oscillator repetition taking the dead time of the detectors into account. However, even when SPDC photons are not injected into the UPC port, the idler-side detector clicks at a rate of 8×10^3 photons/s at 190 mW pump power. Based on the UPC pump power dependence of the rate of this background event, we believe these clicks are due to USPDC photons. For lower pump power, the USPDC photons are mainly contributing the accidental counts. In coincidence measurements, the SPDC pump power was set to be 0.93 mW in order to suppress the accidental counts.

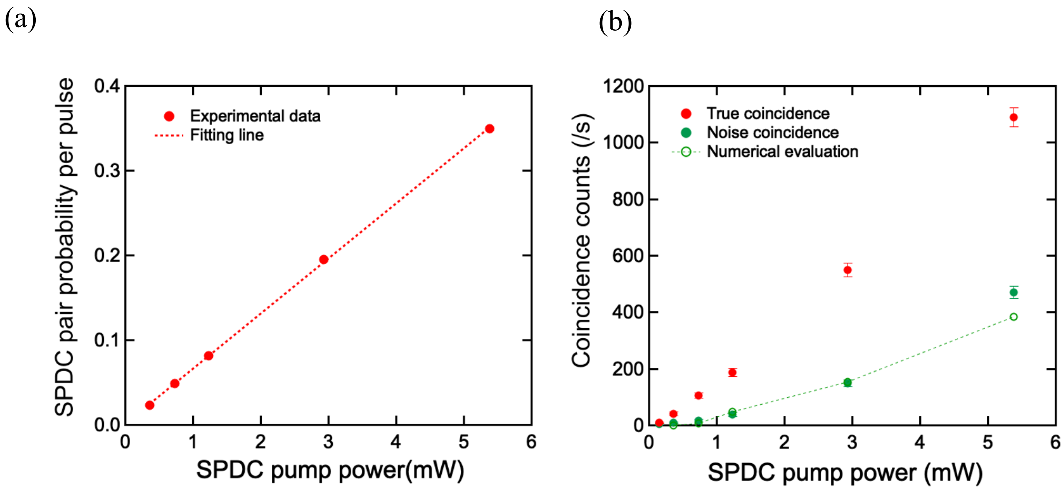

Fig. S2 (a) SPDC pair rate per pulse against SPDC pump power. As the fitting line shows, the rate is linearly dependent on the pump power. (b) True and accidental coincidence against SPDC pump power. The green dashed line and circle is theoretically calculated using the generation rate. The error bars are derived from the standard deviation of the coincidence measurements.

3. Estimation of the up-conversion detector efficiency

We describe the detail of the up-converter which was designed for detecting the infrared idlers. We estimate the conversion efficiency in our experimental condition. Figure S3(a) shows the number of the up-converted idlers against the signal counts at different SPDC pump power values at the UPC pump power of 190 mW. The slope efficiency of the line corresponds to the total quantum efficiency of the detection system, including losses in all optics, which results in the UPC efficiency of 29%.

In addition, we calculate the simulated UPC efficiency using the identical parameter with the experimental setup and investigate the deviation between the experimental and theoretical efficiency curves. The theoretical equation of the conversion efficiency is expressed as follow:

$$\Phi = 2Z_0\gamma^2 I_1 L^2 \operatorname{sinc}^2\left[L\left\{\left(\frac{\Delta k}{2}\right)^2 + 2Z_0\gamma^2 I_1\right\}^{\frac{1}{2}}\right], \qquad (S2)$$

where

$$\gamma = \frac{d_{eff}}{c}\sqrt{\frac{\omega_2\omega_3}{n_2 n_3}} \qquad \text{and} \qquad \Delta k = \left|\overrightarrow{k_1} + \overrightarrow{k_2} - \overrightarrow{k_3} + \overrightarrow{G_\Lambda}\right|,$$

and $\left|\overrightarrow{k_i}\right| = 2\pi n(\lambda_i, T)/\lambda_i$ denotes the wave vector of the pump (i=1), infrared (i=2) and up-converted (i=3) light. Z_0 is the impedance of free space, I_0 is the peak intensity of the UPC pump, and L is the interaction length in the nonlinear crystal. d_{eff} is the effective nonlinear coefficient reduced by the periodic structure. Figure S3(b) shows the theoretical simulation (dashed curve) and experimental values (triangle markers) of the UPC efficiency. The deviation can be attributed to the following two reasons. First, there is the group velocity mismatch between the UPC pump pulse and infrared idlers. According to the Sellmeier dispersion equation, the temporal walk-off induced in the 20 mm-long PPLN is 6 ps between 1.03 μm and 3.81 μm. This walk-off reduced the UPC power to 71.3%. Second, the finite temporal width of the UPC and idler pulses is another factor that reduces the UPC efficiency, which is estimated for continuous waves. The pulse durations of the respective pulses are 4.3 ps and 4.1, as noted in the main text. The intensity at the FWHM of the idlers corresponds to 53% of the peak intensity of the UPC pulse, which indicates that the active power the UPC pump fed in the interaction is reduced to 72%. Taking these two factors into account, the conversion efficiency drops from 61% to 31% with 61% times 0.71 and 0.72, and the experimental results are in good agreement with the theoretical predictions.

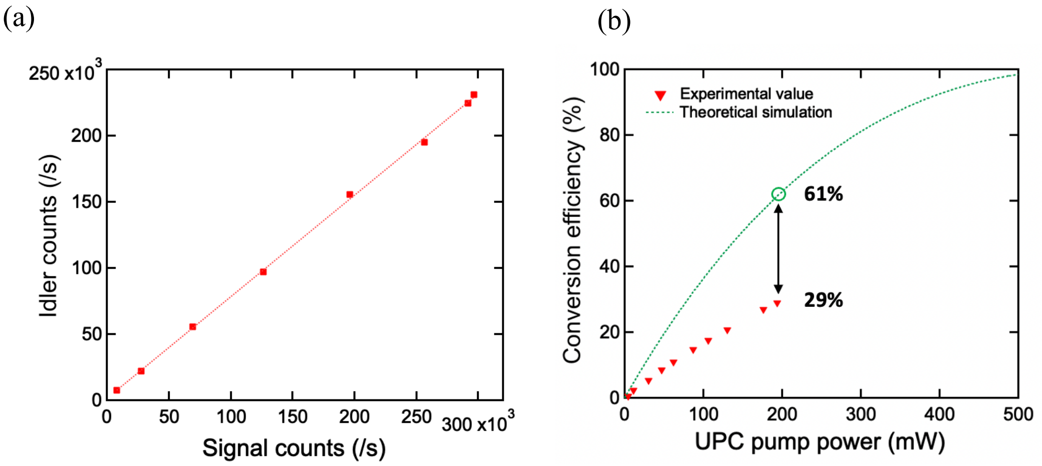

Fig. S3 (a) The up-converted idler counts against the signal counts of the pair 1 at different SPDC pump power. The square dots were the experimental data and the dashed line was a fitting. There was a linear relation between the signal and idler count. The slope corresponded to the ratio of the total quantum efficiency. (b) Theoretical simulation and experimental results of the UPC efficiency. At the maximum power of the pump pulse, each value is 61% and 29%, respectively. The gap between two values is attributed to two reasons, the group velocity mismatch between the UPC pump pulse and infrared idlers and the finite temporal width of the UPC and idler pulses, respectively.

4. Characterization of the UPC in the spectral and temporal domain

Here we specify the spectral window in which the UPC detector efficiently translates the infrared light. Figure S4 plots the normalized UPC efficiency with different spectral widths of the UPC pump pulse of 0.17 THz (in our setup), 0.5 THz, and 0.05 THz, calculated by the equation (S2), which is integrated over each spectral width. The horizontal axis was the wavelength in the infrared region around 3810 nm. The tolerated spectral width is approximately 20 nm, which is almost independent of the UPC spectral width. Assuming the Fourier-transformed pulse, the spectral widths of 0.17, 0.5, and 0.05 THz correspond to pulse durations of 2.6, 0.88, and 8.8 ps, respectively. In the case of 0.5 THz (0.88 ps), the effective crystal length in which the UPC pulse and idlers temporally overlap is reduced to 2 mm, and the conversion efficiency deteriorates significantly in turn. On the other hand, for the spectral width of 0.05 THz (8.8 ps), the peak intensity is reduced to less than half of that for 0.17 THz, which means that the narrow bandwidth reduces the conversion efficiency as well. Moreover, the resulting UPC signals must be extracted with a spectral filter with a linewidth of 1 nm, but such a narrow linewidth reduces the transmittance. This is because there is a trade-off relationship between the tolerant bandwidth and the transmittance of the spectral filter. Such loss reduces the net quantum efficiency of the detection system, which increases the noise counts in coincidence measurements. Therefore, the bandwidth of the UPC pump pulse is optimized at the balance of these trade-offs.

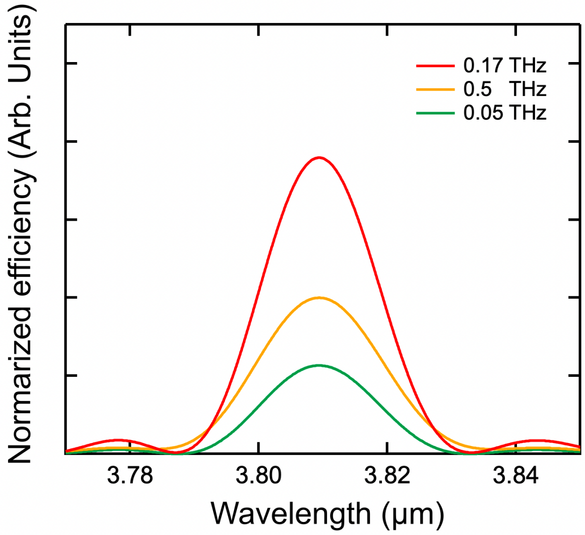

Fig. S4 Spectral window of the UPC detector with the spectral widths of the UPC pump pulse of 0.17 THz, 0.5 THz, and 0.05 THz.

Reference

1. Chong, A., Buckley, J. R., Renninger, W. & Wise, F. All-Normal-Dispersion Femtosecond Fiber Laser. *Opt. Express* **14**, 10095 (2006).
2. Kafka, J. D. & Baer, T. Mode-locked erbium-doped fiber laser with soliton pulse shaping. *Opt. Lett.* **14**, 1269–1271 (1989).
3. Zeng, J. *et al.* Passively synchronized dual-color mode-locked fiber lasers based on nonlinear amplifying loop mirrors. *Opt. Lett.* **44**, 5061 (2019).
4. Kuse, N., Ozawa, A., Nomura, Y., Ito, I. & Kobayashi, Y. Injection locking of Yb-fiber based optical frequency comb. *Opt. Express* **20**, 10509 (2012).
5. Meng, L., Høgstedt, L., Tidemand-Lichtenberg, P., Pedersen, C. & Rodrigo, P. J. Enhancing the detectivity of an upconversion single-photon detector by spatial filtering of upconverted parametric fluorescence. *Opt. Express* **26**, 24712 (2018).
